# Divergence of Peroxisome Membrane Gene Sequence and Expression Between Yeast Species

**DOI:** 10.1534/g3.120.401304

**Published:** 2020-04-21

**Authors:** Claire A. Dubin, Jeremy I. Roop, Rachel B. Brem

**Affiliations:** * Department of Plant and Microbial Biology, UC Berkeley, Berkeley, CA,; ^†^ Fred Hutchinson Cancer Research Center, Seattle, WA,; ^‡^ Berkeley Brewing Science, Inc., Berkeley, CA, and; ^§^ Buck Institute for Research on Aging, Novato, CA

**Keywords:** population genomics, budding yeast, molecular evolution, peroxisomes

## Abstract

Large population-genomic sequencing studies can enable highly-powered analyses of sequence signatures of natural selection. Genome repositories now available for *Saccharomyces* yeast make it a premier model for studies of the molecular mechanisms of adaptation. We mined the genomes of hundreds of isolates of the sister species *S. cerevisiae* and *S. paradoxus* to identify sequence hallmarks of adaptive divergence between the two. From the top hits we focused on a set of genes encoding membrane proteins of the peroxisome, an organelle devoted to lipid breakdown and other specialized metabolic pathways. In-depth population- and comparative-genomic sequence analyses of these genes revealed striking divergence between *S. cerevisiae* and *S. paradoxus*. And from transcriptional profiles we detected non-neutral, directional *cis*-regulatory variation at the peroxisome membrane genes, with overall high expression in *S. cerevisiae* relative to *S. paradoxus*. Taken together, these data support a model in which yeast species have differentially tuned the expression of peroxisome components to boost their fitness in distinct niches.

A central goal of large-scale population genomics is to understand how organisms adapt to their environments, by enabling the search for signatures of natural selection in genome sequences. In humans, for example, the 1000 Genomes project has met with some satisfying successes, as candidate adaptive loci have emerged that had not been detected in smaller samples (Field *et al.* 2016). For organisms with compact genomes and short generation times, sequencing projects of similar scope may lead to an even deeper understanding of the molecular mechanisms of adaptation.

We set out to use budding yeasts as a testbed for large-scale molecular-evolution analysis of population genomes. Despite decades of work in the laboratory, the adaptive history of *Saccharomyces* in the wild remains poorly understood. Previous sequence surveys have reported relatively few signals of positive selection within or between *Saccharomyces* species (Kellis *et al.* 2003; Liti *et al.* 2009; Elyashiv *et al.* 2010; Vishnoi *et al.* 2011; Engle and Fay 2012) (although balancing selection may be more easily detected in this system (Jakobson *et al.* 2019)). To shed more light on the adaptive history of yeast in the wild, we harnessed a recently published compendium of >1000 *S. cerevisiae* strain genomes (Peter *et al.* 2018) to search for loci with marked divergence between this species and its close relative *S. paradoxus*. From among the hits in this scan, we chose a set of genes that function in the peroxisome membrane for a detailed analysis of divergence in sequence and expression, and an inference of the underlying selective forces.

## Methods

### Strains and sequences

Data sources for all analyses are collated in Table S3. For population-genomic analyses of coding regions, we collated sequences from 1011 *S. cerevisiae* strains from (Peter *et al.* 2018) and *S. paradoxus* population-genomic data as follows.

For analyses involving European *S. paradoxus*, we downloaded assemblies and annotation data for *S. paradoxus* from 12 strains from (Bergström *et al.* 2014). We used a custom Python script to extract *S. paradoxus* coding region nucleotide sequences from their annotated location in the genome. For each gene, we aligned the sequences from the strains from a given *S. cerevisiae* population defined in (Peter *et al.* 2018) and all 10 European *S. paradoxus* strains using MUSCLE (Edgar 2004) with the ‘–maxiters’ setting set to 2. We eliminated any sequence consisting of more than 5% gaps, and for the *S. cerevisiae* populations, we did not analyze any with fewer than 10 strains. We used the alignments as input into the *D_XY_* calculation as detailed below.

For analyses involving North American *S. paradoxus*, we downloaded sequence data for the North American A, B, and C subpopulations from (Durand *et al.* 2019). We aligned coding region sequences from each to those of vineyard *S. cerevisiae* from (Peter *et al.* 2018) using MUSCLE with the ‘–maxiters’ setting set to 2 and eliminated any sequence consisting of more than 5% gaps. We discarded any genes with fewer than 300 *S. cerevisiae* strain sequences or any genes with missing *S. paradoxus* sequences in each population, leaving pools of 4285, 3880, and 3868 genes for *S. paradoxus* subpopulations A, B, and C, respectively.

For population-genomic analyses of promoter regions, we downloaded sequence and annotation data for 10 strains of the wine/European *S. cerevisiae* promoter regions defined in (Liti *et al.* 2009). For a given gene we extracted the sequence 500 base pairs upstream of the start codon from each strain, which we defined as the promoter. We likewise extracted the promoter sequence for each gene from each of the 10 European *S. paradoxus* strains from (Bergström *et al.* 2014). We then aligned the complete set of promoter sequences from *S. cerevisiae* and *S. paradoxus* using MUSCLE with the ‘–maxiters’ setting set to 2 and eliminated any sequence consisting of more than 50% gaps or unspecified nucleotides noted as ‘N.’

For coding-region analyses and, separately, promoter analyses using a given population, we discarded any genes for which we had sequence data for fewer than 80% of the respective strains, yielding a total genomic set numbering between 3430 and 3521 genes. We used the alignments as input into the *D_XY_* calculation as detailed below.

For Gene Ontology (GO) enrichment tests as described below, we mapped each gene to its gene ontology groups based on data from geneontology.com (Ashburner *et al.* 2000). We created a pool of genes from which to resample, eliminating dubious ORFs and those mapped only to the broadest GO terms (Molecular Function, Biological Process, or Cellular Component). Genes were annotated as essential or nonessential based on observations in the *S. cerevisiae* type strain (Winzeler *et al.* 1999).

### Sequence analyses

#### Divergence:

We used the alignments of ORF or promoter sequences from a given *S. cerevisiae* and *S. paradoxus* population as input into custom Python scripts to run the *D_XY_* test (Noor and Bennett 2009), using the formula

$$D_{XY}=\frac{1}{n_{x}n_{y}}\overset{n_{x}}{\underset{i=1}{\sum }}\overset{n_{y}}{\underset{j=1}{\sum }}k_{ij}$$

where *n_x_* is the number of *S. cerevisiae* strains, *n_y_* is the number of *S. paradoxus* strains, and *k* is the number of sites with different nucleotides in the same position for each pair of sequences.

To test GO:0005778 for enriched *D_XY_*, for a given *S. cerevisiae* population and *S. paradoxus* population, we calculated *m_true_*, the median *D_XY_* across all *g* genes in the GO term. Next, from the complete set of genes in the genome with alignments and GO annotation (see above), we randomly sampled *g* ORFs or promoters, ensuring the same proportion of essential genes as in the focal term, and calculated the median *D_XY_* of this sample. We repeated this procedure 10,000 times and tabulated the proportion of these resampled groups that yielded a median *D_XY_* greater than or equal to *m_true_*, which we used as a one-sided *p* value assessing the significance of the enrichment of high *D_XY_* in the focal term.

#### Polymorphism:

We used the sequences from the vineyard *S. cerevisiae* population from (Peter *et al.* 2018) and, separately, European *S. paradoxus* from (Bergström *et al.* 2014), as input into a custom Python script to measure π, nucleotide diversity, using the formula

$$\pi =\underset{ij}{\sum }x_{i}x_{j}\pi_{ij}$$

where *x_i_* and *x_j_* are the frequencies of sequences *i* and *j* respectively and $\pi_{ij}$ is the number of nucleotide differences per site in the sequences. The code iterates through each sequence, pairing it with all other sequences and calculates the number of sites with different alleles divided by the total number of sites, returning the sum across all pairs of sequences. As above, we used a resampling test to calculate a one-sided *p* value for elevated median nucleotide diversity in the peroxisome membrane genes compared to resampled cohorts.

#### Phylogenetics:

For phylogenetic analysis of *Saccharomyces* sensu stricto, we downloaded sequence data for *S. cerevisiae* strain S288C, *S. paradoxus* strain CBS432, *S. mikatae* strain IFO1815, and *S. uvarum* strain CBS7001 (Christie *et al.* 2004; Liti *et al.* 2009; Scannell *et al.* 2011). We aligned each open reading frame using PRANK (Löytynoja 2014). For phylogenetic analysis including more *Saccharomycetaceae* species, we downloaded DNA sequences of the 11 closest relatives of *S. cerevisiae* and *S. paradoxus* from (Shen *et al.* 2018) and aligned them using MUSCLE (Edgar 2004) with the ‘–maxiters’ setting set to 2. We used the CodeML module from the PAML package (Yang 2007) to infer branch length for each gene for which we also had data in our population-genomic analyses (see above). The model used an unrooted star tree for analysis of *Saccharomyces* sensu stricto and the tree from (Shen *et al.* 2018) for analysis of the larger species set; in each case we assumed a single protein evolutionary rate. Then, for each species set, for each branch in turn we used the nucleotide branch length as input into a resampling approach as above, and we carried out a one-sided significance test for long branch length in the genes of GO:0005778. The ETE toolkit phylogenetic tree viewer was used to create the trees in Figure S1 (Huerta-Cepas *et al.* 2016).

### Gene expression analysis

To analyze differential expression between *S. cerevisiae* and *S. paradoxus*, we downloaded RNA-seq data sets from (Schraiber *et al.* 2013; Artieri and Fraser 2014), which each profiled *S. cerevisiae* strain S288C, *S. paradoxus* strain CBS438, and their hybrid after growth in rich glucose medium. In the case of (Artieri and Fraser 2014) for each gene we averaged the reported expression from two replicates for each strain in turn. In each data set, for a given gene, we refer to the total differential expression between the species as the log_2_ of expression in purebred *S. cerevisiae*, relative to the analogous quantity in purebred *S. paradoxus*; *cis*-regulatory variation is reported as the log_2_ ratio of allele-specific expression from the two species’ alleles in the hybrid. For each data set, we assessed whether GO:0005778 was enriched for dramatic *cis*-regulatory variation as follows. We used the measurement of *cis*-regulatory variation, for each gene for which we also had data in our population-genomic analyses (see above), as input into a one-sided resampling test as above; we then doubled the resulting *p*-value to yield the result of a two-sided test. We carried out an analogous test for the measurement of total differential expression between the parent species when cultured separately, but we used a one-sided test for elevated expression in *S. cerevisiae*, under the expectation that the direction of differential expression between the species would conform to that of *cis*-regulatory divergence. For concision, only the data from (Schraiber *et al.* 2013) are visualized in Figure 2. For analysis of ribosomal profiling data, we accessed allele-specific ribosomal occupancy in the hybrid and differential ribosomal occupancy between the species when grown separately from (Artieri and Fraser 2014), and analyzed them as above.

In addition, for the analysis of expression profiles of *S. cerevisiae* and other species in Figure S2, we used measurements of *cis*-regulatory variation and total differential expression from (Schraiber *et al.* 2013).

### Data availability

Analysis scripts are available at https://github.com/clairedubin/peroxisome_evolution. Supplemental material available at figshare: https://doi.org/10.25387/g3.12124485.

## Results

### A genome-wide scan for divergence between S. cerevisiae and S. paradoxus:

To identify candidate cases of adaptation in budding yeasts, we first aligned coding sequences from 1011 *S. cerevisiae* strains (Peter *et al.* 2018) and 12 strains of *S. paradoxus* (Bergström *et al.* 2014). We eliminated gap-rich alignments from further analysis, retaining 3664 genes well-suited for molecular-evolution testing. Next, we reasoned that vineyard/wine *S. cerevisiae*, as the most deeply sequenced well-defined population of this species currently identified (Peter *et al.* 2018), would be ideal for exploratory population-genomic analyses. Likewise, the majority of the *S. paradoxus* isolates in our set (10 of 12) were members of a well-defined European population (Bergström *et al.* 2014), suitable for population genomics. For each gene, we tabulated variants between all pairs of vineyard *S. cerevisiae* and European *S. paradoxus*, and we used these counts to calculate the species divergence metric *D_XY_*, a suggestive hallmark of positive selection (Noor and Bennett 2009) (Table S1A).

**Figure 1:**
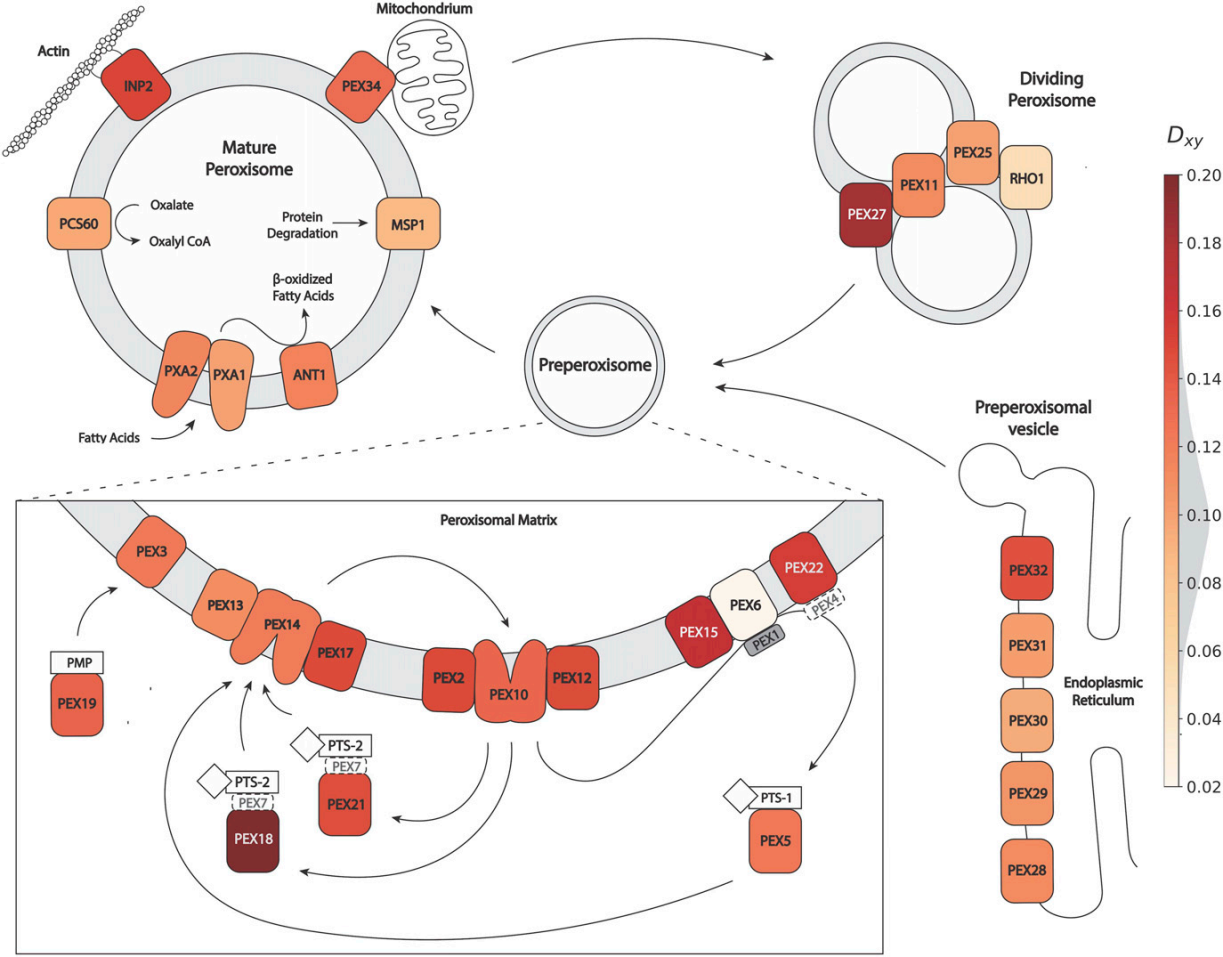
Peroxisome membrane genes are enriched for high *D_XY_*, a population-based metric of sequence divergence, between *S. cerevisiae* vineyard strains and European *S. paradoxus*. Each colored element reports *D_XY_* at one gene of GO:0005778 (peroxisomal membrane), overlaid on a cartoon localization of the encoded protein during peroxisome fission (top right); *de novo* peroxisome biogenesis from the endoplasmic reticulum (bottom right); and function of the mature organelle (left). The inset shows machinery for protein recruitment and import into peroxisomes during maturation. Client proteins are shown in white, as diamonds (with peroxisome targeting signals PTS-1 or PTS-2) or a rectangle (a peroxisomal matrix protein, PMP). Symbols with dashed outlines represent proteins not in GO:0005778. Pex1 (gray) is a member of GO:0005778 but did not have sequence data meeting our quality criteria. Hmg1 and Hmg2 also fall into GO:0005778 but are not shown for clarity (*D_XY_* = 0.087 and 0.092 respectively). The genome-wide distribution of *D_XY_* values is shown superimposed on the color bar legend.

Among the top-scoring genes in this *D_XY_* scan, we noted *PEX18*, a peroxisome membrane-associated protein importer, and *PEX27*, a peroxisome biogenesis factor also resident in the membrane (Table S1A). The potential for evolutionary change between budding yeasts in peroxisome factors was of particular interest given the history of divergence in the function of this organelle across fungi more broadly (Gabaldón 2010). We suspected that other peroxisome factors might also have been subject to divergence between *S. cerevisiae* and *S. para*doxus, and as such we examined the complete set of annotated peroxisome membrane genes in yeast (the Gene Ontology term GO:0005778). Indeed, this group was enriched for high *D_XY_* in our analysis of vineyard *S. cerevisiae* and European *S. paradoxus* (resampling *P* = 0.0227 with all genes beside *PEX18* and *PEX27*, *P* = 0.0069 including all genes; Figure 1). On the strength of this signal, we chose the peroxisome membrane gene cohort for further study of molecular change between the species.

### Species-wide signals of divergence at peroxisome membrane genes

We hypothesized that sequence divergence from *S. paradoxus* at peroxisome membrane genes might not be particular to vineyard *S. cerevisiae*, but instead could reflect a species-wide trend. To test this, we repeated our analysis of *D_XY_* on the peroxisome membrane group using the isolates from each in turn of 23 *S. cerevisiae* populations defined in (Peter, *et al.* 2018), as a comparison against European *S. paradoxus* (Table S1A). We detected elevated *D_XY_* in peroxisome membrane genes in each case (Table 1). Next, by a similar logic, we asked whether the trend for divergence from *S. cerevisiae* would be upheld in analyses of *S. paradoxus* from localities beside Europe. Using three North American *S. paradoxus* populations (Durand *et al.* 2019), we again found striking divergence in the peroxisome membrane gene group (Table S1B). We conclude that the sequences of peroxisome membrane genes are strikingly distinct between the two species, regardless of which isolates and populations we analyze.

**Table 1:** *D_XY_* enrichment among peroxisomal membrane genes

POPULATION	MEDIAN *D_XY_* FOR GO:0005778	p*^a^*
**French Guiana, human**	0.1104	0.0118
**Ale beer**	0.1106	0.0021
**West African cocoa**	0.1114	0.0043
**African palm wine**	0.1112	0.0068
**European wine**	0.1108	0.0069
**European wine subclade 1**	0.1107	0.0073
**European wine subclade 2**	0.1107	0.0083
**European wine subclade 3**	0.1107	0.0089
**European wine subclade 4**	0.1107	0.0089
**Ecuadorean**	0.1097	0.0145
**North American oak**	0.1096	0.0116
**Asian islands**	0.1116	0.0060
**Sake**	0.1112	0.0068
**Asian fermentation**	0.1113	0.0071
**Alpechin**	0.1125	0.0040
**Brazilian bioethanol**	0.1107	0.0044
**French dairy**	0.1109	0.0047
**African beer**	0.1119	0.0031
**Mosaic beer**	0.1107	0.0064
**Mixed origin**	0.1096	0.0066
**Mosaic region 1**	0.1101	0.0083
**Mosaic region 2**	0.1107	0.0051
**Mosaic region 3**	0.1105	0.0071

Each row reports the enrichment among peroxisomal membrane genes (GO:0005778) for elevated *D_XY_*, a population-based metric of sequence divergence, in a comparison of the indicated *S. cerevisiae* population and European *S. paradoxus*. In a given population, the median *D_XY_* across all genes in the genome is between 0.0971 and 0.0988.

a Resampling-based significance from a test for elevated *D_XY_* using strains of the indicated *S. cerevisiae* population.

### Directional cis-regulatory divergence at peroxisome membrane genes

**Figure 2:**
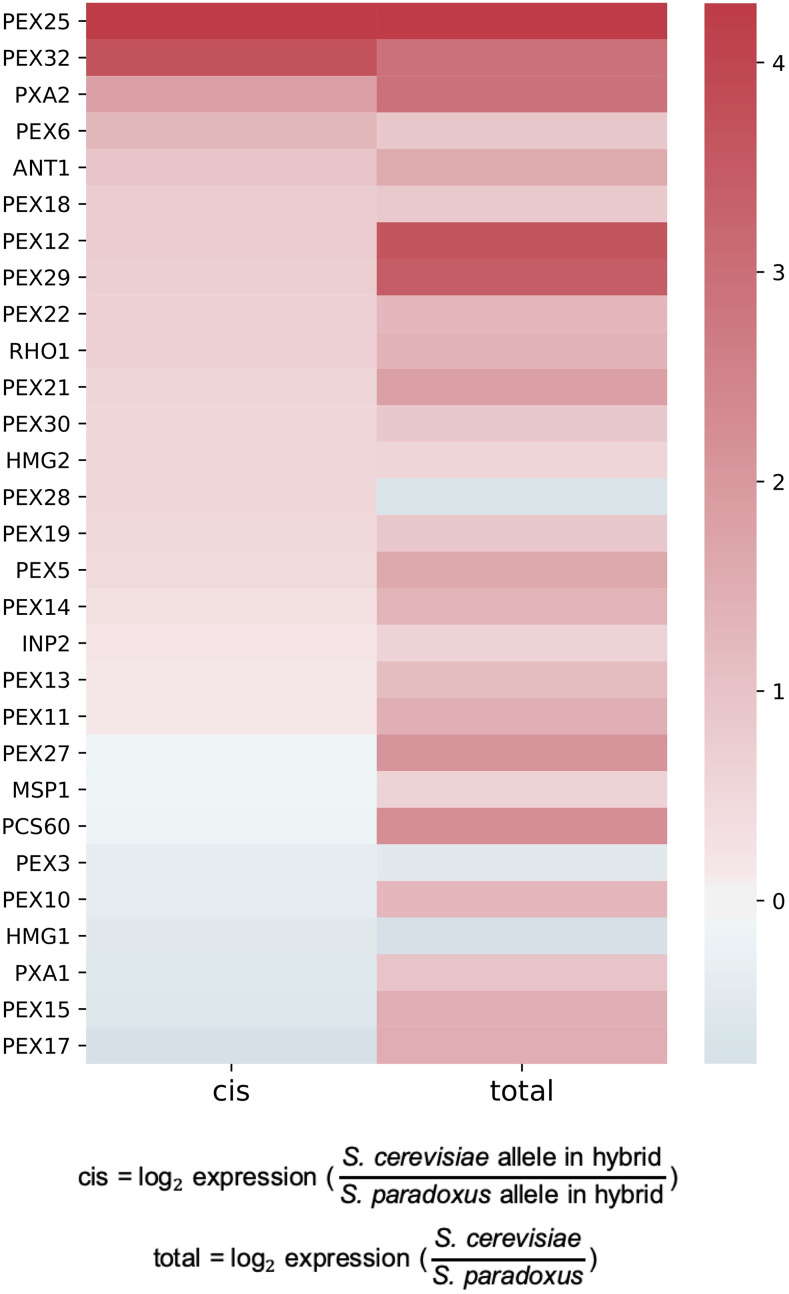
*S. cerevisiae* expresses peroxisome membrane genes more highly than does *S. paradoxus*, as a product in part of *cis*-regulatory changes. Each cell reports expression, as a ratio between *S. cerevisiae* and *S. paradoxus*, of the indicated peroxisome membrane gene from GO:0005778. Total, expression measured in purebred species; *cis*, expression from the indicated species’ allele in a diploid interspecific hybrid, reflecting effects of *cis*-regulatory divergence.

We next aimed to investigate the possible molecular mechanisms of peroxisome membrane gene evolution. We found no detectable signal in tests of non-neutral amino acid substitution rates (data not shown). As such, we instead hypothesized that evolution could have tuned the regulation of this gene cohort differently between *S. cerevisiae* and *S. paradoxus*. To evaluate this, we made use of our laboratory’s test for *cis*-regulatory variants that drive expression in the same direction across unlinked genes of a pathway, a pattern unlikely under neutrality (Bullard *et al.* 2010). We applied this scheme to two independently collected profiles of *cis*-regulatory divergence between *S. cerevisiae* and *S. paradoxus* in rich glucose medium, each measured from allele-specific expression in the interspecies hybrid (Schraiber *et al.* 2013; Artieri and Fraser 2014). The results revealed significant directional *cis*-regulatory divergence in the peroxisomal membrane group (resampling *P* = 0.0062 and 0.0320, respectively, from the two data sources), with the *S. cerevisiae* allele of each gene tending to drive higher expression relative to the *S. paradoxus* allele (Figure 2).

We also inspected profiles of total expression divergence between the species—the product of DNA sequence variants acting both in *cis* and in *trans*, as measured from expression in purebreds. Using this quantity we detected directional change in peroxisome membrane genes with higher expression in *S. cerevisiae*, as was true in our analysis of *cis*-regulatory variation (Figure 2; resampling *P* = 0.0035 and 0.0275, respectively, from the two data sources). Furthermore, in ribosomal profiling data (Artieri and Fraser 2014), we detected higher ribosomal occupancy of peroxisomal membrane genes in cultures of *S. cerevisiae* relative to *S. paradoxus*, attesting to the relevance of the mRNA expression difference between the species in terms of protein abundance (though no such imbalance could be detected between the alleles in the hybrid; resampling *P =* 0.0171 and 0.7754, respectively). We conclude that, with growth conditions held constant, *S. cerevisiae* and *S. paradoxus* are hard-wired to express peroxisome membrane genes differently, as a function in part of a suite of *cis*-regulatory changes at the unlinked gene loci.

We reasoned that many of the elements underlying *cis*-acting expression variation between the species in our gene cohort would likely fall in promoters. Yet the initial evidence of sequence divergence that we had noted for the peroxisome membrane genes was in our *D_XY_* test applied to coding regions (Table 1 and Table S2). We expected that, if such a signal were the consequence of linked selection on adaptive variants in promoters, the latter would mirror what we had detected in open reading frames, and exhibit significant divergence. Consistent with this prediction, we detected high *D_XY_* in the promoters of the peroxisome membrane genes, in a comparison of the European *S. cerevisiae* and European *S. paradoxus* populations (resampling *P* = 0.0019). Thus at a sizeable window centered on a given gene in the cohort, encompassing both the upstream region and the open reading frame, strains of the two species are robustly and recurrently different from one another in terms of sequence. Together, this trend and our expression-based test support a model in which the regulation of the peroxisome membrane genes has been subject to different selective pressures in *S. cerevisiae* and *S. paradoxus*.

### Inferring a history of evolutionary volatility at peroxisome membrane genes

In principle, divergence in peroxisome membrane genes could reflect a history in which an ancestral state was established before the divergence of *S. cerevisiae* and *S. paradoxus*, followed by stabilizing selection in one species and directional selection in the other. Alternatively, the peroxisome membrane gene group may have been evolutionarily more volatile, undergoing changes in multiple Saccharomycete lineages. To investigate this, we pursued phylogenetic analyses of DNA sequence across species, using a small set of *Saccharomyces* sensu stricto species (Christie *et al.* 2004; Liti *et al.* 2009; Scannell *et al.* 2011) and, separately, a larger set of *Saccharomycetaceae* (Shen *et al.* 2018). These tests revealed an excess of sequence variation in the peroxisome membrane genes along each branch of the phylogeny, significantly so in most cases (Figure S1). Likewise, expression profiles in rich glucose medium (Schraiber *et al.* 2013) revealed a regulatory program in the distant relative *S. uvarum* that was distinct from the expression levels of either *S. cerevisiae* or *S. paradoxus* (Figure S2). The latter suggests an evolutionary event in the *S. uvarum* lineage unrelated to the changes between our two focal species. These data preclude the straightforward inference of a state of the peroxisome membrane group ancestral to *Saccharomyces* sensu stricto or those species farther out, and reflect a likely history of evolutionary volatility over millions of years.

## Discussion

In this work, we have harnessed the wealth of population-genomic data available for *S. cerevisiae* (Peter *et al.* 2018) in tests of divergence between this species and *S. paradoxus*. To date, hits from such scans have been at a premium, which may reflect the limited statistical power associated with smaller yeast genome cohorts (Kellis *et al.* 2003; Elyashiv *et al.* 2010; Vishnoi *et al.* 2011; Engle and Fay 2012; Bergström *et al.* 2014). Indeed, most studies of adaptive loci in *Saccharomyces* have found their gene candidates by methods other than molecular-evolution surveys (Will *et al.* 2010; Engle and Fay 2012; Martin *et al.* 2012; Fraser *et al.* 2012; Roop *et al.* 2016; Weiss *et al.* 2018; Duan *et al.* 2019). By contrast, the strong molecular-evolution signal we report here attests to the power of the deep population-sampling approach.

Our analysis has centered on a pattern of sequence divergence from *S. paradoxus* across hundreds of *S. cerevisiae* isolates in peroxisome membrane genes. Given that we have used the absolute divergence measure *D_XY_* rather than metrics that normalize divergence by within-species polymorphism, our conclusions are not contingent on any effects of the latter (and the peroxisome membrane gene group was not an outlier with respect to polymorphism in any case; resampling *P* = 0.68 and *P* = 0.25 for vineyard *S. cerevisiae* and European *S. paradoxus*, respectively). Rather, we interpret the elevated *D_XY_* signal as an indicator of a history of positive selection on the gene cohort in one or both species, although strictly speaking it could also be consistent with balancing selection in the ancestor (Guerrero and Hahn 2017).

Our findings dovetail with the widespread divergence in peroxisomal function across the fungal kingdom in general, which has perhaps been facilitated by the ease of gains and losses of peroxisomal localization signals in protein sequences (Yanagida *et al.* 2015). Apart from its conserved role in fatty acid oxidation, the peroxisome has evolved in particular fungi to carry out methanol catabolism; antibiotic, siderophore, and biotin biosynthesis; and a wound-healing function in filamentous species (Gabaldón 2010; Maruyama and Kitamoto 2013; Lim and Keller 2014). However, our data do not afford any insight into possible biochemical changes in the function of this organelle between *S. cerevisiae* and *S. paradoxus*. Instead, the strongest mechanistic inference we can make derives from our analysis of gene expression: *S. cerevisiae* and *S. paradoxus* have differentially tuned when and how peroxisome membrane genes come on, in a pattern inconsistent with neutrality as a driving force. It is tempting to speculate that the high expression by *S. cerevisiae* could reflect a historical need to boost fatty acid metabolism in its niche. As no conclusions about niche or ecology can be drawn from our genomics approach, future ecological and phenotypic analyses relevant to the peroxisome will be of prime interest.

A key conclusion from our findings is that variants in many peroxisome membrane genes—with regulatory changes at promoters likely of particular importance—came together to build an evolutionary innovation as *S. cerevisiae* and *S. paradoxus* diverged. Assuming that these changes represent an adaptation to an environmental challenge, we can infer that no single Mendelian locus was sufficient to solve the ecological problem at hand. This complexity would be consistent with the deleterious effects of overexpressing individual peroxisome membrane genes on their own in yeast (Elgersma *et al.* 1997; Sopko *et al.* 2006). As population sequencing data sets grow, such instances of polygenic adaptation (Pritchard and Di Rienzo 2010) may prove to be the norm, in systems from microbes to mammals.
